# Innovative breakthroughs facilitated by single-cell multi-omics: manipulating natural killer cell functionality correlates with a novel subcategory of melanoma cells

**DOI:** 10.3389/fimmu.2023.1196892

**Published:** 2023-06-26

**Authors:** Zhijie Zhao, Yantao Ding, Lisa Jia Tran, Gang Chai, Li Lin

**Affiliations:** ^1^ Department of Plastic and Reconstructive Surgery, Shanghai Ninth People’s Hospital, Shanghai Jiao Tong University School of Medicine, Shanghai, China; ^2^ Shanghai Jiao Tong University School of Medicine, Shanghai, China; ^3^ Department of Dermatology, The First Affiliated Hospital, Institute of Dermatology, Anhui Medical University, Hefei, Anhui, China; ^4^ China Key Laboratory of Dermatology, Ministry of Education, Anhui Medical University, Hefei, Anhui, China; ^5^ Department of General, Visceral, and Transplant Surgery, Ludwig-Maximilians-University Munich, Munich, Germany

**Keywords:** single-cell sequencing, novel biomarker, tumor heterogeneity, cancer immunotherapy, melanoma, clinical outcome

## Abstract

**Background:**

Melanoma is typically regarded as the most dangerous form of skin cancer. Although surgical removal of *in situ* lesions can be used to effectively treat metastatic disease, this condition is still difficult to cure. Melanoma cells are removed in great part due to the action of natural killer (NK) and T cells on the immune system. Still, not much is known about how the activity of NK cell-related pathways changes in melanoma tissue. Thus, we performed a single-cell multi-omics analysis on human melanoma cells in this study to explore the modulation of NK cell activity.

**Materials and methods:**

Cells in which mitochondrial genes comprised > 20% of the total number of expressed genes were removed. Gene ontology (GO), gene set enrichment analysis (GSEA), gene set variation analysis (GSVA), and AUCcell analysis of differentially expressed genes (DEGs) in melanoma subtypes were performed. The CellChat package was used to predict cell–cell contact between NK cell and melanoma cell subtypes. Monocle program analyzed the pseudotime trajectories of melanoma cells. In addition, CytoTRACE was used to determine the recommended time order of melanoma cells. InferCNV was utilized to calculate the CNV level of melanoma cell subtypes. Python package pySCENIC was used to assess the enrichment of transcription factors and the activity of regulons in melanoma cell subtypes. Furthermore, the cell function experiment was used to confirm the function of TBX21 in both A375 and WM-115 melanoma cell lines.

**Results:**

Following batch effect correction, 26,161 cells were separated into 28 clusters and designated as melanoma cells, neural cells, fibroblasts, endothelial cells, NK cells, CD4+ T cells, CD8+ T cells, B cells, plasma cells, monocytes and macrophages, and dendritic cells. A total of 10137 melanoma cells were further grouped into seven subtypes, i.e., C0 Melanoma BIRC7, C1 Melanoma CDH19, C2 Melanoma EDNRB, C3 Melanoma BIRC5, C4 Melanoma CORO1A, C5 Melanoma MAGEA4, and C6 Melanoma GJB2. The results of AUCell, GSEA, and GSVA suggested that C4 Melanoma CORO1A may be more sensitive to NK and T cells through positive regulation of NK and T cell-mediated immunity, while other subtypes of melanoma may be more resistant to NK cells. This suggests that the intratumor heterogeneity (ITH) of melanoma-induced activity and the difference in NK cell-mediated cytotoxicity may have caused NK cell defects. Transcription factor enrichment analysis indicated that TBX21 was the most important TF in C4 Melanoma CORO1A and was also associated with M1 modules. *In vitro* experiments further showed that TBX21 knockdown dramatically decreases melanoma cells’ proliferation, invasion, and migration.

**Conclusion:**

The differences in NK and T cell-mediated immunity and cytotoxicity between C4 Melanoma CORO1A and other melanoma cell subtypes may offer a new perspective on the ITH of melanoma-induced metastatic activity. In addition, the protective factors of skin melanoma, STAT1, IRF1, and FLI1, may modulate melanoma cell responses to NK or T cells.

## Introduction

Melanoma is generally considered a fatal kind of skin cancer, predominantly caused by cell-intrinsic age-related mutations (1, 2). Elderly patients with melanoma have worse prognoses and response rates to targeted treatment than younger melanoma patients (3). Due to its tendency for metastasis, skin melanoma is generally considered the worst type of skin cancer. Even though melanoma may be efficiently treated by surgical excision of *in situ* lesions, the metastatic illness remains challenging to cure.

Prior to the beginning of this decade, chemotherapy was the standard treatment method for metastatic melanoma (4). However, chemotherapy was generally ineffective, mostly palliative, and limited in prolonging survival due to its resistance to apoptosis (5). Melanoma is particularly favorable for immune treatment due to its high immunogenicity.

Recent advances in targeted and immunotherapeutic treatments have introduced substantial changes in the standard treatment for advanced stages of melanoma, known as metastatic disease. Cancer therapies have greatly progressed thanks to an increased emphasis on the immune system. Utilizing immune checkpoint inhibitors (ICIs), which concentrate on T cells capable of recognizing malignancies, is one of the essential advances (6). The introduction of immunotherapy, more specifically ICIs, has introduced a great change in the environment. Ipilimumab, a monoclonal antibody (mAb) targeting the cytotoxic T-Lymphocyte Antigen 4 (CTLA-4), was the first ICI approved by the FDA in 2011. It increased the overall response rate to 19% and the percentage of patients who survived for 5 years to 20% (7). Additionally, the introduction of anti-programmed cell death protein-1 (anti-PD-1) further enhanced the percentage of 5-year survival rates to 30-40% (8–10). However, more than half of the patients who were treated with anti-PD-1 promptly gained resistance to treatment. Also, it has been reported that around 30% of patients who initially react well to anti-PD-1 therapy eventually acquire resistance to the drug (11, 12), thus suggesting that other immunotherapies should be researched and developed (13). Natural killer (NK) cell immunotherapy is thought to have substantial promise and therapeutic prospects in the immunotherapy of melanoma; it arises as a promising frontier for future melanoma treatment, necessitating additional scientific scrutiny and clinical validation to ensure its safety and efficacy.

NK cells, as lymphocytes of the innate immune system, hold essential importance and play a crucial role in antitumor responses (14). NK cells can detect tumors resistant to T-cell lysis due to deletion or downregulation of MHC class I antigens, hence complementing anticancer action. Consequently, it has been proposed that activating NK cells against malignant cells is a useful technique for tumor treatment, in addition to T-cell immunotherapy (15, 16). NK cells can immediately target changed cells while protecting healthy nonmalignant cells. This is accomplished by initiating cell death or releasing granules containing gran-zyme B and perforin. Apoptosis is induced by the activation of receptors (17). Thus, NK cells hold promise for cancer immunotherapy, and a variety of approaches, including antibodies that block inhibitory receptors (e.g., NKG2A (18), TIGIT (19), are being developed to enhance NK cell-mediated tumor immunity. Multiple studies have reported that individuals with colorectal carcinoma, gastric cancer, and lung cancer with high amounts of NK cells in their malignant tissue are more likely to have a better prognosis (20, 21). More importantly, NK cells may be effective effector cells in the treatment of melanomas because of the low surface levels of HLA class I molecules on melanoma cells and the fact that these cells frequently produce ligands (such as B7H6, MICA/B, ULBPs, PVR, and nectin2) recognized by key activating NK receptors (22). However, although NK cells were found to be active toward melanoma cells *in vitro*, multiple studies have shown impaired NK cell function in melanoma patients (23). It has been hypothesized that this ineffectiveness was caused by the high level of immunosuppression produced in the tumor microenvironment (TME) due to the presence of both cancer cells and stromal cells.

Consequently, restoring NK cell function, which results in a greater response to melanoma, could be potentially effective for individuals who do not react to conventional metastatic melanoma treatments. However, the heterogeneity and differences of NK cell-related pathway activity in metastatic melanoma tissue are not well understood. Accordingly, we performed a single-cell RNA-seq study of human metastatic melanoma cells.

## Materials and methods

### Data of single cell source collection and processing

**Figure 1:**
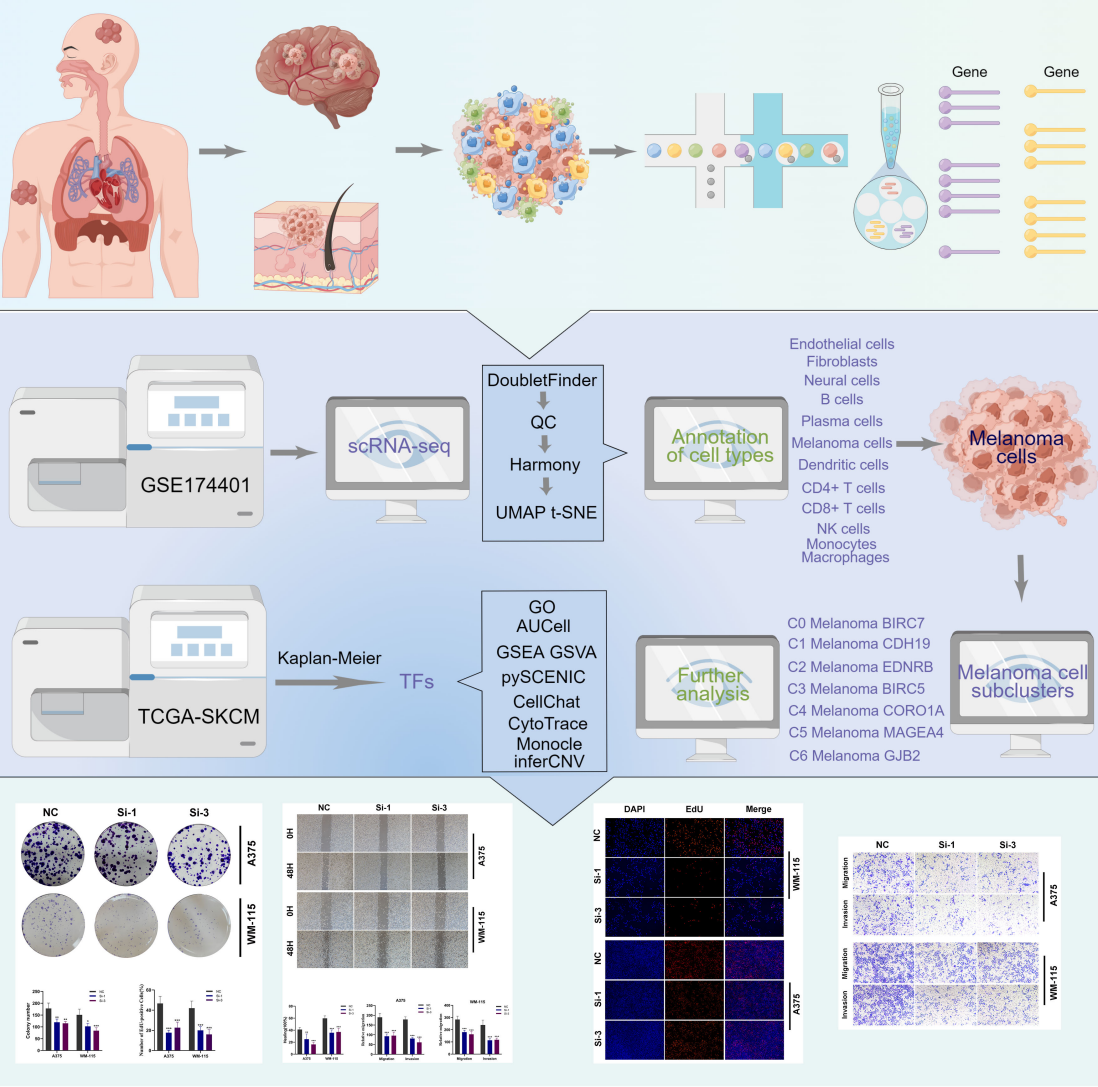
The analysis workflow for this research.

The overall analysis workflow for this research was shown in **Figure 1**. The scRNA-seq data of melanoma skin metastases, melanoma brain metastases (MBM), and leptomeningeal melanoma metastases (LMM) were downloaded from GSE174401 via the Gene Expression Omnibus database (https://www.ncbi.nlm.nih.gov/geo/). The Seurat package (v4.1.1) was used to load the 10X genomics data from each individual sample into R software (4.1.3). Firstly, the DoubletFinder program (v2.0.3) was utilized to detect any doublets that may have occurred as a consequence of cell encapsulation or as a result of random pairings of cells and samples that were not separated during the production of the samples. In this study, we excluded low-quality cells exhibiting fewer than 500 identified genes or more than 6000 genes. It is crucial to emphasize that we eliminated cells displaying an expression of mitochondrial genes exceeding twenty percent. Genes exhibiting expression in fewer than three cells were also excluded from the following analysis. This research did not require ethical approval for our research since we used data from a publicly accessible database.

### Clustering and cell type identification of scRNA-seq

In order to execute the natural log transformation, the log(x+1) method was employed to compute the amount of gene expression in each cell as a fraction of the gene multiplied by 10,000. The normalized expression matrix was used to find the top 2000 highly variable genes (HVGs). Then, we scaled them before running a principal component analysis (PCA) on these genes. Using the R Harmony package (version 1.0) (24), batch effects were eliminated based on the top 50 PCA components. Based on harmony-corrected data, k-nearest neighbors (KNN) were calculated, and a shared nearest neighbor (SNN) graph was constructed. The modular function was then modified based on the clustering algorithm to accomplish cluster recognition. The identified clusters were presented on the 2D map made with the t-distributed stochastic neighbor embedding (tSNE) or uniform manifold approximation and projection (UMAP) for dimension reduction method (25).

Using the “FindAllMarkers” function, we identified the marker genes for each cluster based on the following parameters: logfc.threshold = 0.25, min.pct = 0.25, and min.diff.pct = 0.25. The DotPlot and featureplot tools of Seurat were used to show the expression patterns of each marker gene across clusters. Based on the DEGs and well-known cellular markers mentioned in the scientific literature (26), the cell groups were annotated. Additionally, to further investigate melanoma cell heterogeneity, the melanoma cells were re-clustering. Each subgroup of melanoma cells was then labeled based on its distinctive genes.

### Gene ontology (GO), enrichment analysis by gene set enrichment analysis (GSEA), gene set variation analysis (GSVA), and AUCell analysis of differentially expressed genes (DEGs) among melanoma subtypes

We compared the upregulated genes to the related terms according to the Gene Ontology (GO) database (http://www.geneontology.org/) to determine the functional significance of the DEGs; GO terms with varying degrees of enrichment were determined using hypergeometric testing. The likelihood-ratio test was used to identify genes that were differentially expressed in different types of melanoma cells. In this research, the p-values were changed to account for the FDR, and it was decided that a p-value of 0.05 was statistically significant. GSEA software (version 4.1.0) was used to investigate gene function using the MSIGDB database from the GSEA website (http://software.broadinstitute.org/gsea/msigdb). To determine which pathways differ the most between subgroups, the differential induction of genes was utilized to rank them, and the Pi package (version 2.6.0) and MsigdbH were employed to conduct a gene set enrichment analysis. The majority of pathway studies were conducted on the 50 hallmark pathways outlined in the molecular signature database (27). Furthermore, using the data of melanoma cells, we also evaluated the activity of NK cell-related pathways. Next, to assign pathway activity estimates to individual cells, we used the GSVA package (version 1.42.0) (28). AUCell (29) is a novel approach for identifying cells with active genes in single-cell RNA-seq data. A gene set is the input to AUCell, and the output is the “activity” of the gene set in each cell. To functionally annotate genes, annotation and visualization of GO terms was used by metascape (30) (http://metascape.org).

### Cell-cell communication

Based on single-cell RNA sequencing data, the CellChat package (version 1.4.0) projected cell–cell communication across all cell types (31). To predict cell-cell interactions among the different cell types, a significance threshold of 0.05 (P-value) was employed.

### Pseudotemporal ordering of melanoma cells

The pseudotime trajectories of melanoma cells were analyzed using the Monocle package (version 2.22.0). Using pseudotemporal profiling of scRNA-seq data, Monocle aims to identify cellular alterations that occur during differentiation of melanoma cells. After inserting the scale of raw UMI counts into the “newCellDataSet” function with its clustering information, it was transformed into a reduced dimensional space using the discriminative dimensionality reduction with trees (DDRTree) technique, a more current manifold learning method. According to pseudotime, melanoma cells were then arranged. The plot pseudotime heatmap was used to identify and display the genes whose expression varied simultaneously with pseudotime.

### CytoTrace analysis of melanoma cells

CytoTRACE (version 0.3.3) (32) is a computational framework for recreating the relative differentiation state of single-cell RNA sequencing data using the gene expression profile. The differentiation state of cells may be deduced from scRNA-seq data without any previous knowledge. Initially, a KNN graph containing information on the undirected relationships between cells was built. CytoTRACE was then utilized to determine the recommended time order of cells. The KNN graph and planned time were then utilized to generate a transfer matrix, which was afterwards shown on a UMAP scatter plot.

### Single cell copy number variation (CNV) evaluation of tumor cells

InferCNV (version 1.12.0) were utilized to calculate the CNV level. Using copy-kat, copy-number karyotyping of aneuploid tumors was created to differentiate between non-malignant and malignant cell types. The NK cell was utilized as a reference in inferCNV to identify whether or not other cancer cells exhibit substantial chromosomal copy number variation.

### Scenic analysis

SCENIC is a tool that uses scRNA-seq data to reconstruct gene regulatory networks while identifying stable cell states. Using the package pySCENIC (version 0.10.0) in Python (version 3.7), this research assessed the enrichment of transcription factors and the activity of regulons. The gene regulatory network was developed using co-expression and DNA motif analysis as the foundation. The cell state was identified by examining the activity of the network in each cell. The gene-motif ranking (10 kb around the transcription start site) was used as a guide to determine the search space around the transcription start site for transcription factor regulatory networks. Human gene-motif rankings are collected from https://resources.aertslab.org/cistarget/. Hallmark Gene Set from Molecular Signatures Database (MsigDB) was utilized as the database (33).

### Kaplan–Meier survival curves of selected genes

Bulk transcriptomic data of TCGA- SKCM cohort from the TCGA database (https://portal.gdc.cancer.gov/). Among them, 404 melanoma samples with complete clinical information were used for further analysis. According the expression of the selected genes, patients were divided into two groups (gene high, gene low, by means). In order to assess the survival disparity, Kaplan–Meier survival curves were plotted first. For survival analysis, the survival package (version 3.4.0) and the survminer package (version 0.4.9) were employed (34), and TFs with differing survival rates between gene high group and gene low group were selected.

Subsequently, the univariate Cox regression analysis was utilized to further filter for TFs linked with the outcomes of melanoma patients (p < 0.05). To narrow the range of TFs, Least Absolute Shrinkage and Selection Operator (LASSO) regression was conducted using 10-fold cross-validation and a p-value of 0.05 (35). TFs with prognostic values were included in the LASSO regression analysis, and receiver operating characteristic (ROC) curves of 1, 3, and 5 years were drawn using the timeROC package to estimate the efficiency of these TFs (version 0.4.0).

Finally, by correcting for confounding factors (stage, age, and gender), the multivariate Cox regression analysis was used to determine independent protection or risk factors among the selected TFs.

### Cell lines culture of melanoma cells and cell transfection

A375 and WM-115 melanoma cell lines were purchased from the Cell Resource of the Shanghai Life Sciences Institute. These cells were grown in 10% fetal bovine serum (FBS), 1% streptomycin, and penicillin (Gibco BRL, USA) in MEM or RPMI-1640 (Gibco BRL, USA) (Gibco, Invitrogen, Waltham, MA, USA). The cells were cultured at 37°C, 5% CO2.

Small interfering RNA (siRNA) constructs were used to achieve TBX21 knockdown (GenePharma, Suzhou, China). In addition, TBX21 siRNA sequences (Si-1 and Si-3) are listed in **Table S1** of the **Supplementary Materials**. In brief, cells were seeded at 50% confluence in a 6-well plate and then infected with negative control (NC) and knockdown (Si-1 and Si-3). Every transfection was performed with Lipofectamine 3000. (Invitrogen, USA).

### Extraction of RNA and real-time PCR

TRIzol was used to extract total RNA from cell lines according to the manufacturer’s instructions (15596018, Thermo). cDNA was then made utilizing the PrimeScript TMRT kit (R232-01, Vazyme). The real-time polymerase chain reaction (RT-PCR) was performed using SYBR Green Master Mix (Q111-02, Vazyme), and the expression levels of each mRNA were standardized to the level of mRNA GAPDH. Expression levels were quantified using the 2^–ΔΔCT^ method. Tsingke Biotech (Beijing, China) supplied all primers, and the primer sequences are detailed in **Supplemental Table S1**.

### Cell counting kit-8, colony formation, and EdU experiments

The cell suspension was seeded at a density of 3×10^3^ cells per well in 96-well plates. After adding 10 μL of CCK-8 labeling agent (A311-01, Vazyme) to each well, the plate was incubated for 2 hours at 37°C in the dark. Absorbance at 450 nm was measured at 0, 24, 48, 72, and 96 hours using an enzyme-labeled meter (A33978, Thermo) to evaluate cell viability. Each well of a 6-well plate contained 1×10^3^ transfected cells, and we maintained cell growth for 14 days. Two PBS washes and 15 minutes in 4% paraformaldehyde were performed before staining with Crystal Violet (Solarbio, China). Stained with 0.1% crystal violet. The experiment was performed after the cells had adhered to the wall using a 96-well plate with 2×10^4^ treated cells in each well. The 5-Ethynyl-2’-deoxyuridine (EdU) assay was then performed according to the manufacturer’s instructions (Ribobio, China). Cell proliferation was quantified using an inverted microscope.

### Wound-healing assay and transwell assay

Transfected cells were seeded in 6-well plates and grown in a cell incubator until they reached 95% confluence. Each culture well was scraped with a sterile 200μL plastic pipette tip, and unattached cells and detritus were rinsed twice with PBS. After collecting photographs of the scratch wounds at 0 h and 48 h, we utilized the Image J program to measure the breadth of the scratches. Transwell assay was utilized for cell invasion and migration experiments. Forty-eight hours were spent incubating treated A375 and WM-115 (2×10^5^) in the upper chamber of 24-well plates. The top of the plate was either precoated with matrigel solution (BD Biosciences, USA) or left untreated in order to evaluate the cells’ capacity to invade and migrate. After removing the cells from the top surface, the residual cells on the bottom layer were fixed with 4% paraformaldehyde and stained with 0.1% crystal violet (Solarbio, China).

## Results

### Quality control, normalization, and removal of a batch effect

First, doublets were detected using DoubletFinder (version 2.0.3) and eliminated prior to further analysis. Subsequently, we removed cells that expressed < 500 genes, or > 20% of the total number of expressed genes, as mitochondrial genes in our study. Low-quality cells with < 200 genes, < 3 cells, or > 6000 genes found were also eliminated. The Harmony package (version 0.1.0) was used to eliminate batch effects between samples based on the top 50 PCA components.

### Cell type annotation of 28 clusters

**Figure 2:**
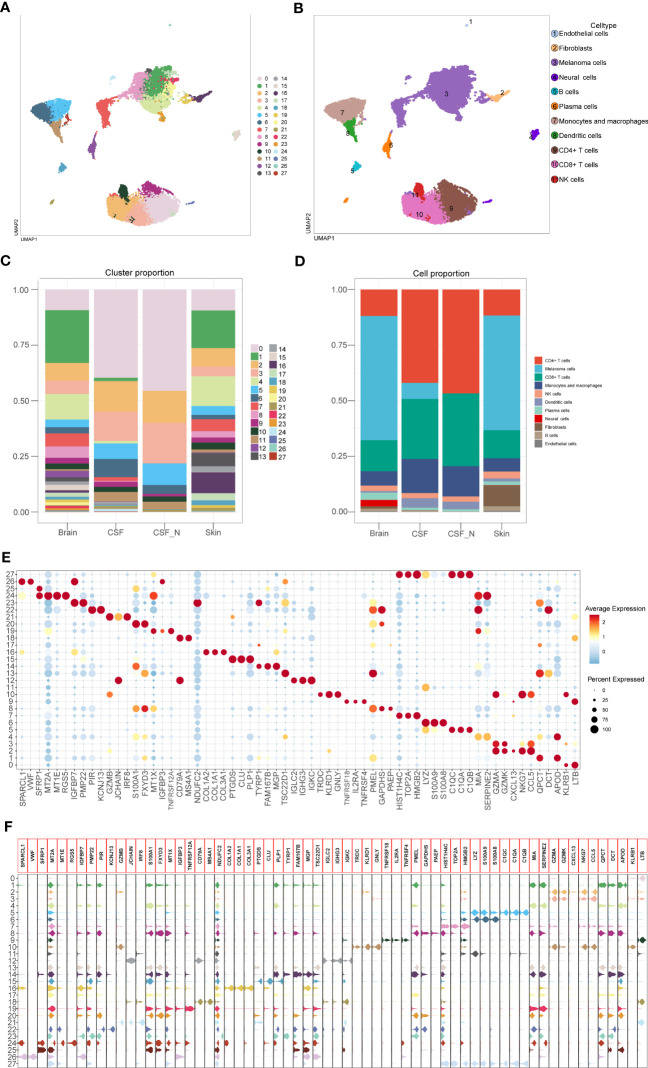
Identification of the cell types based on specific markers among 28 clusters. **(A)** The 2D plots of UMAP dimensionality reduction of the 26,161 high-quality cells. **(B)** The 2D plots of UMAP dimensionality reduction of cell type. **(C, D)** The percentage of clusters and cell types between tissues was represented on a proportion chart. **(E, F)** Dot plot and violin plot showing top3 marker genes for 28 clusters.

After batch effect correction, 26,161 cells were divided into 28 clusters with 1.0 resolution. According to the specific expression genes of 28 clusters, these clusters were labeled as melanoma cells (cluster 1, 4, 7, 8, 13, 14, 17, 19, 20, 22, 23, and 24), neural cells (clusters 15 and 25), fibroblasts (cluster 16), endothelial cells (cluster 26), NK cells (cluster 10), CD4+ T cells (clusters 0, 9), CD8+ T cells (clusters 2 and 3), B cells (cluster 18), plasma cells (clusters 12 and 21), monocytes and macrophages (clusters 5, 6, 27), and dendritic cells (cluster 11). We used the UMAP techniques to decrease dimensionality, and then plotted the result as a 2D scatter plot (**Figures 2A, B**). The proportion chart depicted the percentages of clusters and cell types between samples (**Figures 2C, D**). Dot plot and violin plot were used to depict the expression of specific markers among clusters (**Figures 2E, F**). From the aforementioned cell type statistics, we determined that the proportion of various cell types varied significantly between different tissues.

### Identification of melanoma cell subtypes

**Figure 3:**
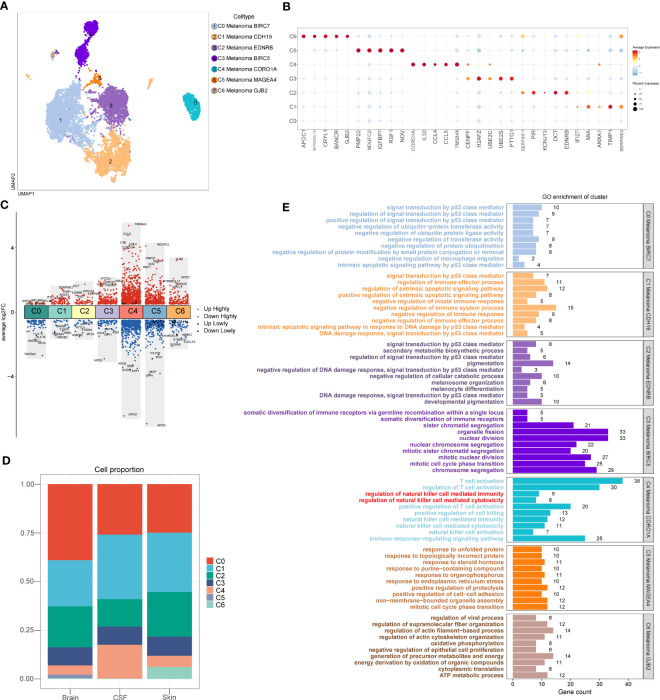
Melanoma cell subtypes. **(A)** The 2D plots of UMAP dimensionality reduction of the 10,137 melanoma cells. **(B)** Dot plot showing top5 marker genes for melanoma cell subtypes. **(C)** Columnar scatter plot of DEGs among melanoma cell subtypes (a value of adjusted p-value < 0.01 is displayed in red (up) or blue (down), whereas a value of adjusted p-value≥ 0.01 is displayed in black). **(D)** The percentage of melanoma cell subtypes between tissues was represented on a proportion chart. **(E)** GO enrichment analysis of DEGs among melanoma cell subtypes.

A total of 10137 melanoma cells were clustered into 7 subtypes (C0 Melanoma BIRC7, C1 Melanoma CDH19, C2 Melanoma EDNRB, C3 Melanoma BIRC5, C4 Melanoma CORO1A, C5 Melanoma MAGEA4, C6 Melanoma GJB2) (**Figure 3A**). Dot plot was used to depict the expression of markers among melanoma cell subtypes (**Figure 3B**). In addition, the columnar scatter plot of DEGs among melanoma cell subtypes is shown in **Figure 3C**. In addition, the proportion chart depicted the percentages of melanoma cell subtypes between tissues (**Figure 3D**).

### The heterogeneity and immune-related pathways of melanoma cell subtypes

According to the analysis of GO (**Figure 3E**), the DEGs of C4 Melanoma CORO1A were primarily enriched in NK cell-mediated cytotoxicity, NK cell activation, regulation of NK cell-mediated immunity, regulation of NK cell-mediated cytotoxicity, NK cell-mediated immunity, T cell activation, regulation of T cell activation, positive regulation of T cell activation, positive regulation of cell killing, and immune response−regulating signaling pathway. However, compared to the C4 Melanoma CORO1A, the DEGs of other melanoma cell subtypes were mainly enriched in positive regulation of signal transduction by p53 class mediator, negative regulation of macrophage migration, positive regulation of extrinsic apoptotic signaling pathway, negative regulation of innate immune response, negative regulation of immune system process, negative regulation of immune response, negative regulation of immune effector process, mitotic cell cycle phase transition, regulation of actin filament−based process.

**Figure 4:**
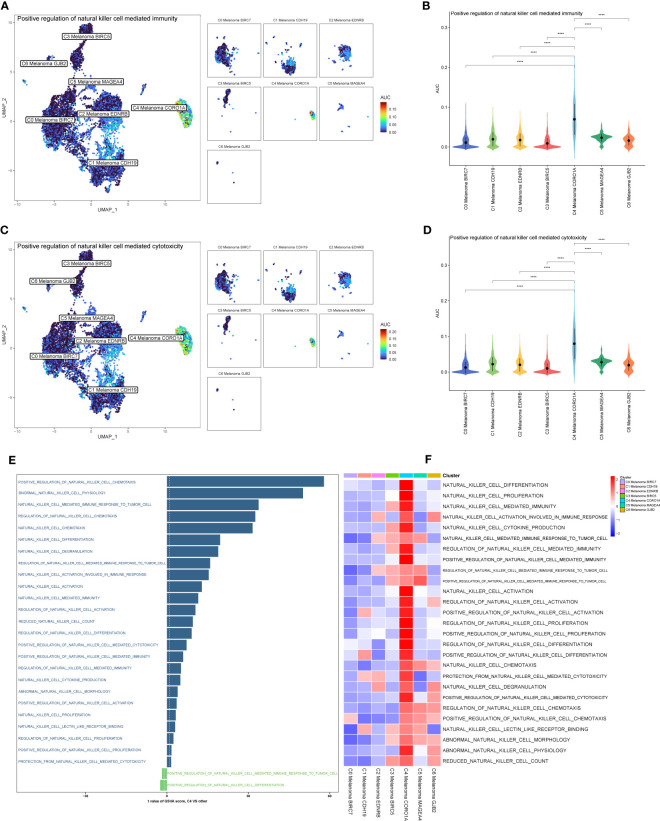
AUCell, GSVA, and GSEA analysis of NK cell-related genes among melanoma cell subtypes. **(A, B)** Differences in positive regulation of NK cell-mediated immunity activities scored per cell by AUCell among melanoma cell subtypes. **(C, D)** Differences in positive regulation of NK cell-mediated cytotoxicity activities scored per cell by AUCell among melanoma cell subtypes. **(E)** GSVA enrichment analysis between C4 Melanoma CORO1A and other melanoma cell subtypes. **(F)** GSEA enrichment analysis among melanoma cell subtypes. ****P < 0.0001.

**Figure 5:**
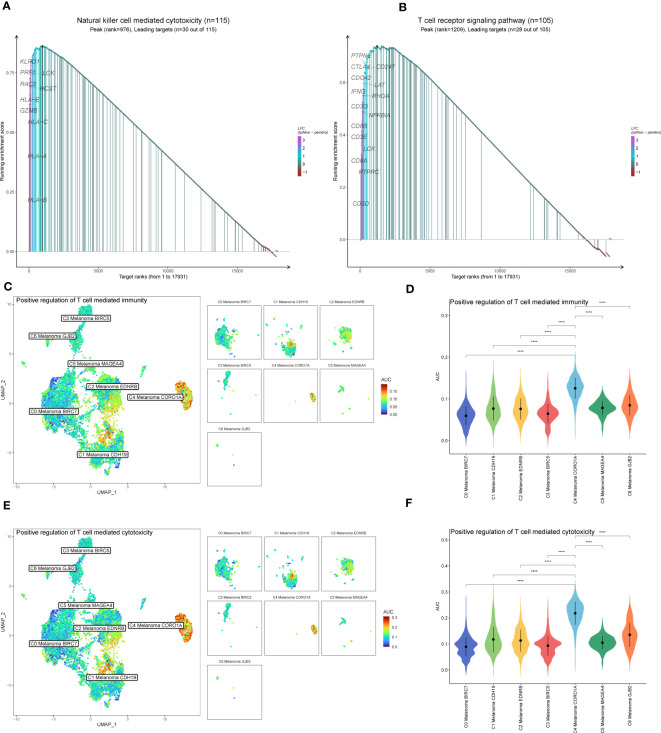
GSEA and AUCell analysis among melanoma cell subtypes. **(A)** GSEA enrichment analysis of NK cell-mediated cytotoxicity among melanoma cell subtypes. **(B)** GSEA enrichment analysis of T cell receptor signaling pathway among melanoma cell subtypes. **(C, D)** Differences in positive regulation of T cell-mediated immunity activities scored per cell by AUCell among melanoma cell subtypes. **(E, F)** Differences in positive regulation of T cell-mediated cytotoxicity activities scored per cell by AUCell among melanoma cell subtypes. ****P < 0.0001.

The results above of GO enrichment across melanoma cell subtypes may imply that the regulation of NK and T cell-mediated cytotoxicity-associated signal pathways are active in C4 melanoma CORO1A compared to other melanoma cell subtypes. Thus, in order to compare the differences in positive regulation of NK (**Figure 4**) and T cell (**Figure 5**) mediated immunity among melanoma cell subtypes, the AUCell GSVA and GSEA were used for further analysis. The results of AUCell indicated that compared to the other melanoma cell subtypes, the positive regulation of NK cell-mediated immunity and cytotoxicity was significantly activated in C4 Melanoma CORO1A (**Figures 4A–D**). In addition, the results of AUCell also revealed that the positive regulation of T cell-mediated immunity and cytotoxicity was significantly activated in C4 Melanoma CORO1A (**Figures 5C–F**).

In addition to the above-reported data, the results of GSVA were also used to examine the differences between C4 Melanoma CORO1A and other subtypes of melanoma cells in terms of NK cell-mediated cytotoxicity-related pathways (**Figure 4E**). In a nutshell, consistent with the prior analysis, the results of GSEA revealed that NK cell-mediated immunity-related signal pathways were more active in C4 Melanoma CORO1A than in other subtypes of melanoma cells (**Figure 4F**). Besides, the results of GSEA analysis of NK cell-mediated cytotoxicity and T cell receptor signaling pathway between C4 Melanoma CORO1A and other melanoma cell subtypes are shown in **Figures 5A, B**. In conclusion, the above-reported data suggested that the activity of NK cell-mediated cytotoxicity is upregulated in C4 Melanoma CORO1A.

### Potential communication networks between NK cells and melanoma cell subtypes

**Figure 6:**
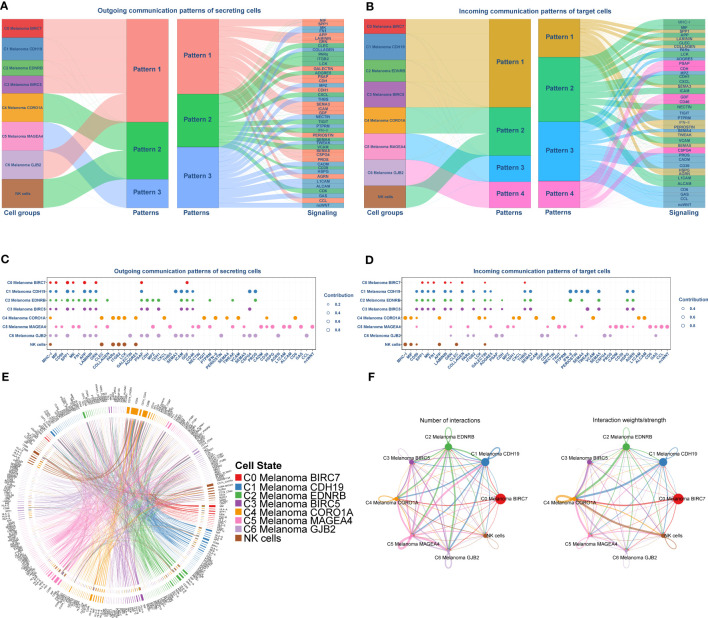
Cell-Chat analysis between NK cells and subtypes of melanoma cells. **(A, B)** Sankey Diagram of communication patterns of secreting cells between NK cells and subtypes of melanoma cells. **(C, D)** Dot plot of communication patterns of secreting cells between NK cells and subtypes of melanoma cells. **(E)** Chordal graphs of the overall ligand–receptor pairs. **(F)** The numbers (left) and weights (right) of ligand receptors among all NK cells and subtypes of melanoma cells.

The above enrichment analysis results indicated NK cell-related pathways were much more active in C4 Melanoma CORO1A than in other subtypes of melanoma cells. Accordingly, uncovering the potential difference in molecular connections between NK cells and melanoma cell subtypes is important. Therefore, the CellChat package (version 1.4.0) was employed to determine the potential molecular interactions between NK cells and melanoma cell subtypes and ligand-receptor pairings. This was done to establish communication networks between NK cells and melanoma cell subtypes. The overall results of the CellChat analysis are shown as a Sankey diagram, dotplot, and chordal graphs in **Figure 6**. Further investigation of outgoing communication among these cells revealed that NK cells and C4 Melanoma CORO1A belong to the same pattern (**Figures 6A, B**). In addition, detailed information regarding potential molecular interactions was presented in **Figures 6C–F**.

### Pseudotime trajectories analysis of melanoma cell subtypes

**Figure 7:**
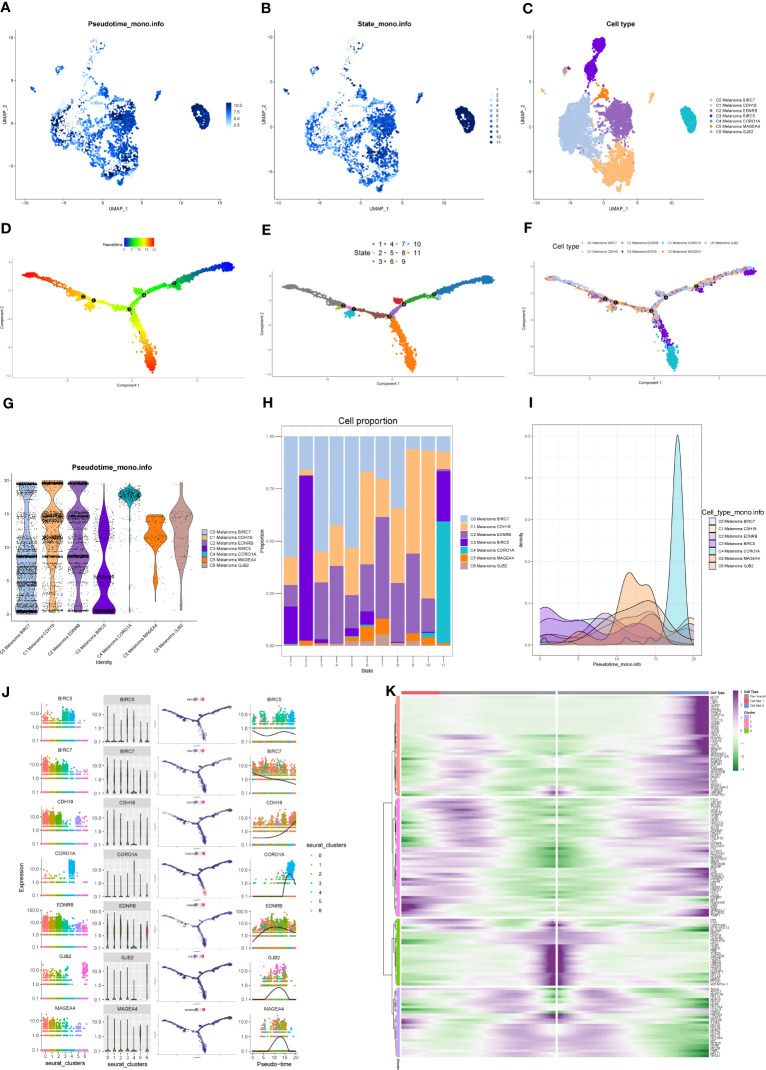
Pseudotime trajectories analysis of melanoma cell subtypes. **(A–C)** The UMAP plots show pseudotime trajectories of subtypes of melanoma cells. **(D–F)** The trajectory of differentiation of subtypes of melanoma cells predicted by Monocle. **(G–I)** The percentage of subtypes of melanoma cells between different states shown in a violin plot, proportion chart, and ridgeline plot. **(J)** The expression of BIRC7, EDNRB, BIRC5, CORO1A, MAGEA4, and GJB2 among different states and subtypes. **(K)** Heatmap depicting the scaled expression of top 20 markers of melanoma cell subtypes in two branches, as classified into four major gene clusters.

Monocle package (version 2.22.0) of R was applied to evaluate the differentiation of melanoma cell subtypes. The results of Monocle showed that compared to the other melanoma cell subtypes, C4 Melanoma CORO1A had the highest differentiation (**Figures 7A–I**). The expression changes in melanoma cell subtype core markers (BIRC7, CDH19, EDNRB, BIRC5, CORO1A, MAGEA4, and GJB2) are shown in **Figure 7J**. Besides, the top 20 markers of melanoma cell subtypes are also shown in heatmaps based on the pseudotime trajectories analysis (**Figure 7K**).

### The AUCell analysis of positive regulation of epithelial-to-mesenchymal transition and oxidative phosphorylation among melanoma cell subtypes

The results of AUCell indicated that compared to the other melanoma cell subtypes, the positive regulation of epithelial-to-mesenchymal transition, oxidative phosphorylation related genes were significantly suppressed in C4 Melanoma CORO1A (**Supplemental Figure 1**).

### CytoTrace, cell stemness, and inferred CNV analysis of melanoma cell subtypes

**Figure 8:**
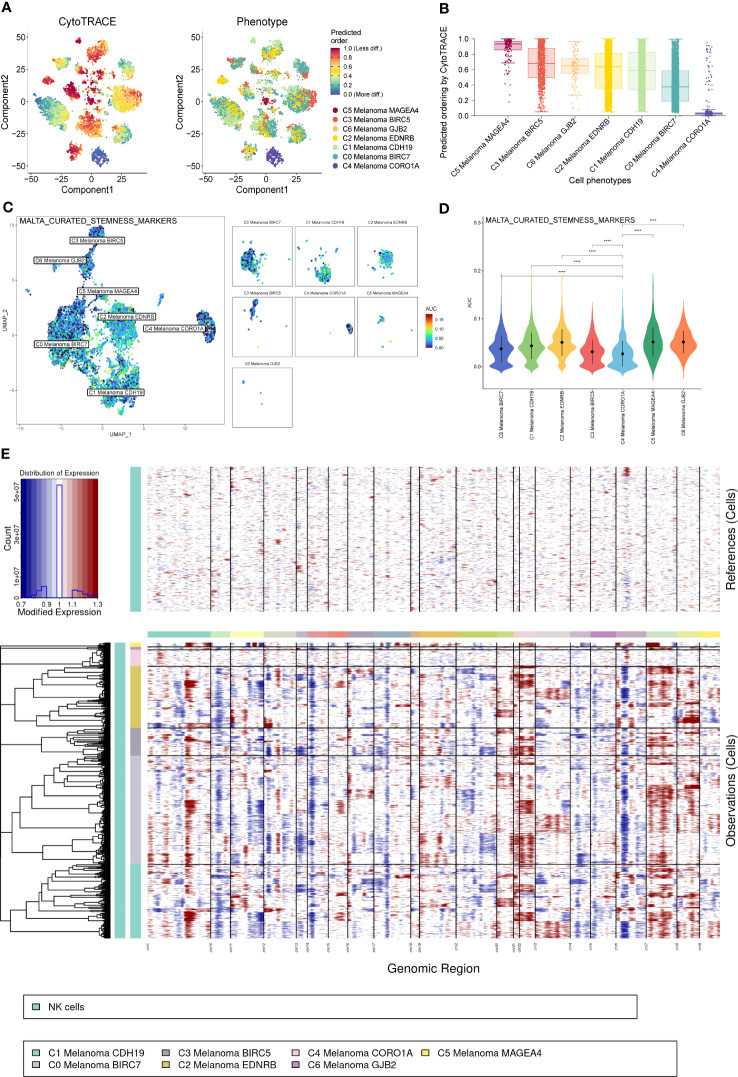
CytoTrace and inferred copy number variation (CNV) analysis of melanoma cell subtypes. **(A)** Differentiation of melanoma cell subtypes. **(B)** The lowest differentiation of C5 Melanoma MAGEA4 and the highest differentiation of C4 Melanoma CORO1A can be observed from the graph. **(C, D)** Differences in cell stemness scored per cell by AUCell among melanoma cell subtypes, C4 Melanoma CORO1A has the lowest cell stemness. **(E)** The heatmap displayed large-scale CNVs of melanoma cells. The red color represents a high CNV level, and the blue represents a low CNV level. ****P < 0.0001.

Consistent with previous results of pseudotime trajectories analysis, the results of CytoTrace showed that compared to the other melanoma cell subtypes, C4 Melanoma CORO1A had the highest differentiation (**Figures 8A, B**).

In order to elucidate the cell stemness of melanoma cell subtypes, AUCell was applied to score the degree of cell stemness in the melanoma cell subtypes (**Figures 8C, D**). The results of AUCell showed that C4 Melanoma CORO1A had the lowest cell stemness compared to the other melanoma cell subtypes.

**Figure 9:**
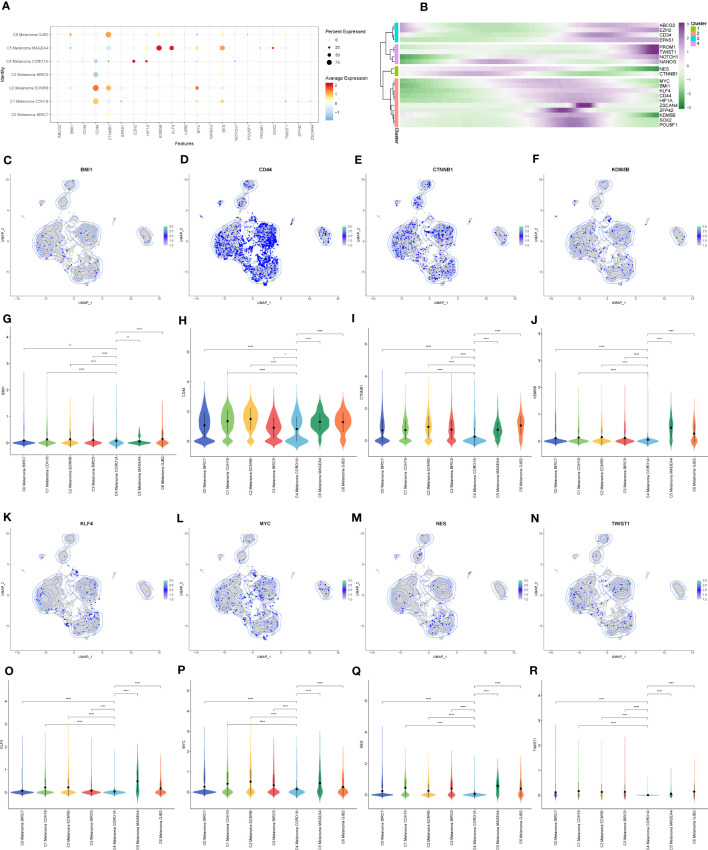
The expression of cell stemness-related genes in melanoma cell subtypes. **(A)** The dot plot showed the expression of cell stemness-related genes. **(B)** Heatmap depicting the scaled expression of cell stemness-related genes of melanoma cell subtypes, as classified into four major gene clusters. **(C–R)** The UMAP plots and violin plots show the differential expression of cell stemness-related genes among subtypes of melanoma cells. *P < 0.05, **P < 0.01, ****P < 0.0001.

Next, InferCNV analysis was used to infer the CNV status of cells from different cell types using NK cells as controls (**Figure 8E**). C4 Melanoma CORO1A was characterized by their unique CNV amplification/deletion on different chromosomes compared with other melanoma cell types. Furthermore, the dot plot, heatmap, UMAP plots, and violin plots were used to show the expression of cell stemness-related genes among subtypes of melanoma cells (**Figure 9**).

### The AUCell analysis of positive regulation of NK and T cell activation-related genes in subtypes of melanoma cells

**Figure 10:**
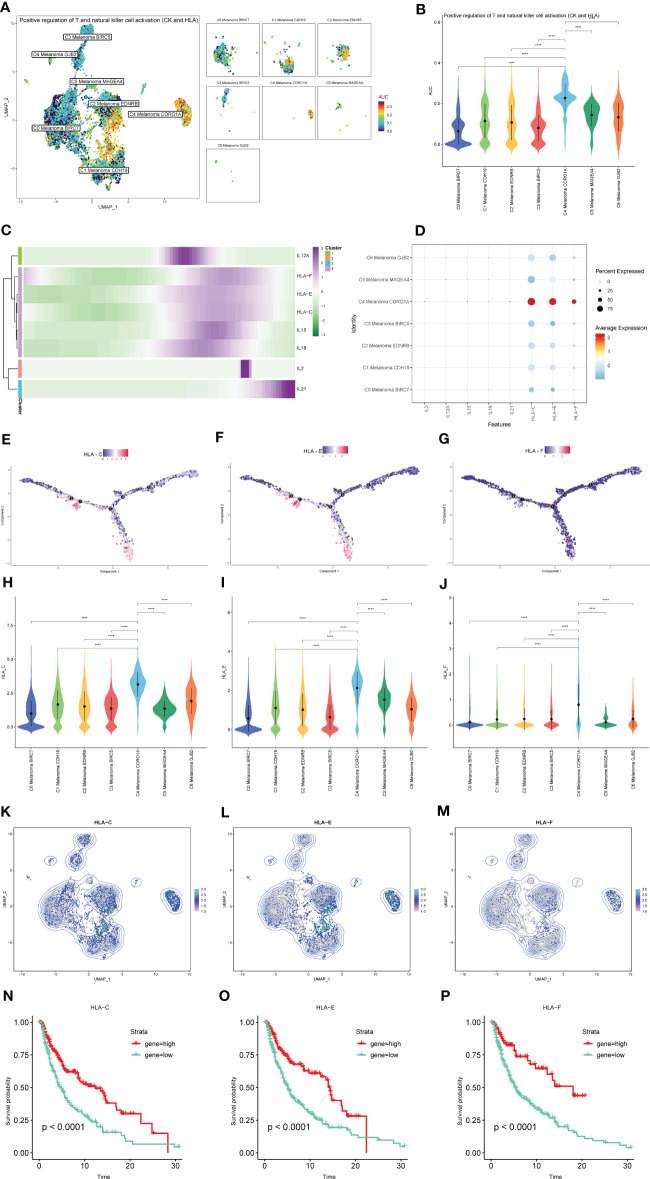
The expression of positive regulation of NK and T cell activation-related genes in subtypes of melanoma cells. **(A, B)** Differences in positive regulation of NK and T cells activation-related genes activities scored per cell by AUCell among melanoma cell subtypes. **(C)** Heatmap depicting the scaled expression of positive regulation of NK and T cells activation-related genes of melanoma cell subtypes, as classified into four major gene clusters. **(D)** The dot plot shows the expression of positive regulation of NK and T cells activation-related genes. **(E–G)** The expression of HLA-C, HLA-E, and HLA-F in pseudotime trajectories of subtypes of melanoma cells. **(H–M)** The UMAP plots and violin plots show the differential expression of HLA-C, HLA-E, and HLA-F among subtypes of melanoma cells. **(N–P)** Kaplan-Meier survival plots of the high and low gene groups for HLA-C, HLA-E, and HLA-F. ****P < 0.0001.

The results of AUCell indicated that compared to the other melanoma cell subtypes, the positive regulation of NK and T cell activation-related genes were significantly activated in C4 Melanoma CORO1A (**Figures 10A, B**). The dot plot, heatmap, UMAP plots, and violin plots were used to show the expression of positive regulation of NK and T cell activation-related genes among subtypes of melanoma cells (**Figures 10C–M**). The above results suggest that most of these genes are expressed at the end of the differentiation. Consistent with the results of pseudotime trajectories analysis, compared with other subtypes of melanoma cells, these genes had the higher expression in C4 Melanoma CORO1A. In addition, the Kaplan-Meier survival analysis further revealed that the high-expression groups of HLA-C, HLA-E, and HLA-F had a longer survival time among patients with metastatic melanoma (**Figures 10N–P**).

### Gene regulatory network analysis of melanoma cell subtypes

**Figure 11:**
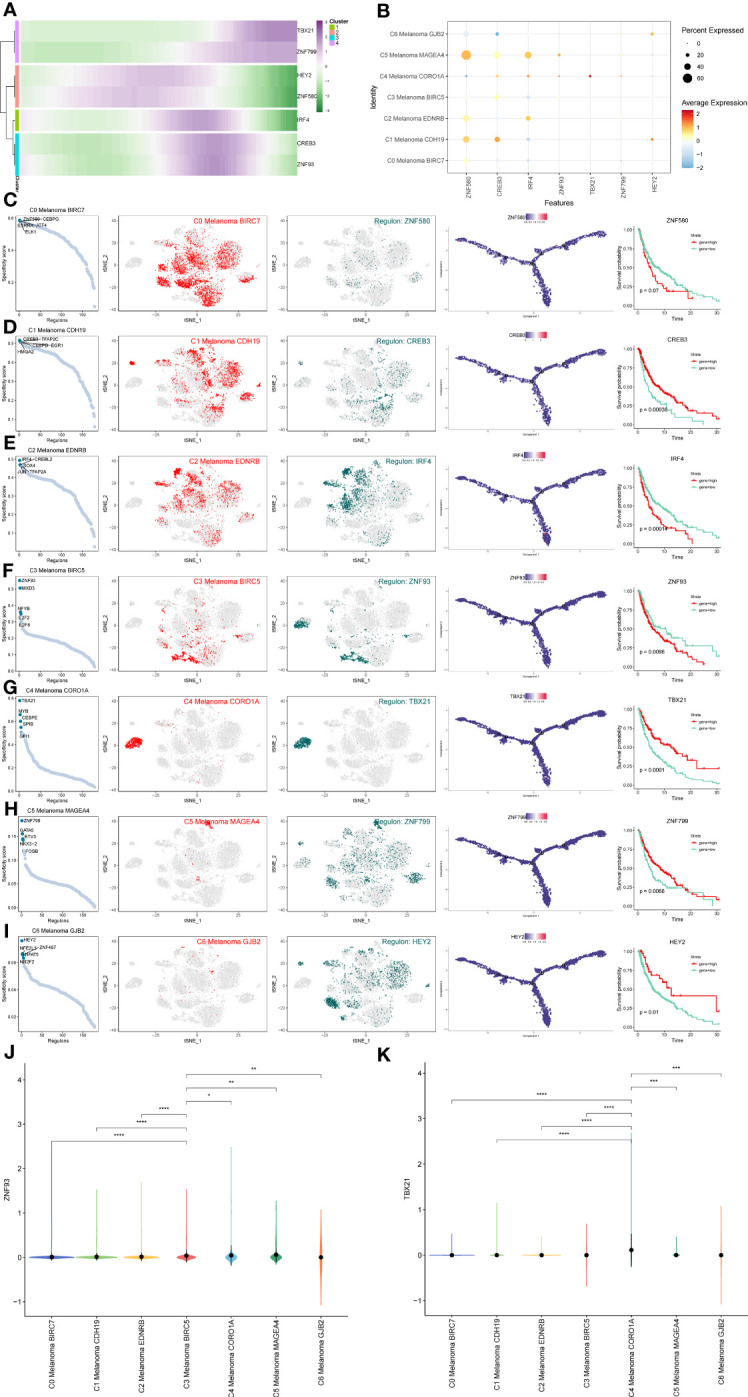
Gene regulatory network analysis of melanoma cell subtypes. **(A)** Heatmap depicting the scaled expression of TBX21, ZNF799, HEY2, ZNF580, IRF4, CREB3, and ZNF93 among melanoma cell subtypes, as classified into four major gene clusters. **(B)** The dot plot shows the expression of these TFs. **(C–I)** Rank for regulons in melanoma cell subtypes based on regulon specificity score (RSS). Melanoma cell subtypes are highlighted in the t-SNE (red dots). Binarized regulon activity scores (RAS) for the top regulon of melanoma cell subtypes on t-SNE (perform Z score normalization across all samples, and set 2.5 as the cutoff to convert to 0 and 1) (dark green dots). Kaplan-Meier survival plots of the 7 key TFs of melanoma cell subtypes. **(J, K)** The violin plots show the expression of ZNF93 and TBX21 in melanoma cell subtypes. *P < 0.05, **P < 0.01, ***P < 0.001, ****P < 0.0001.

In order to determine the core TFs detectable in subtypes of melanoma cells, a SCENIC analysis was carried out. The pySCENIC was used to infer the gene regulatory networks of all melanoma cell subtypes. The most activated TFs of these melanoma cells subtypes, as determined by the results of the study on cell type-specific regulon activity, included ZNF580 (C0 Melanoma BIRC7), CREB3 (C1 Melanoma CDH19), IRF4 (C2 Melanoma EDNRB), ZNF93 (C3 Melanoma BIRC5), TBX21 (C4 Melanoma CORO1A), ZNF799 (C5 Melanoma MAGEA4), and HEY2 (C6 Melanoma GJB2) (**Figure 11**). The heatmap was used to demonstrate the expression levels of the genes TBX21, ZNF799, HEY2, ZNF580, IRF4, CREB3, and ZNF93 during the pseudotime trajectories of four distinct subtypes of melanoma cells (**Figure 11A**).

In order to more intuitively display gene expression, the dot plot was also used (**Figure 11B**). Regulons in melanoma cell subtypes were ranked according to their specificity score (RSS). In the t-SNE, melanoma cell subtypes were highlighted (red dots). Binarized regulon activity scores (RAS) for the leading regulon of melanoma cell subtypes based on t-SNE (conduct Z score normalization across all samples and use 2.5 as the cutoff to convert 0 and 1) (dark green dots) were also shown. Kaplan-Meier survival graphs were made for melanoma cell subtypes’ seven most important TFs (**Figures 11C–I**). These results suggest that the patients with high expression of TBX21, which is the top-regulated TF of C4 Melanoma CORO1A, have longer survival probabilities. In addition, the expression of ZNF93 and TBX21 in C4 melanoma CORO1A most significantly differed between other melanoma subtypes (**Figures 11J, K**).

**Figure 12:**
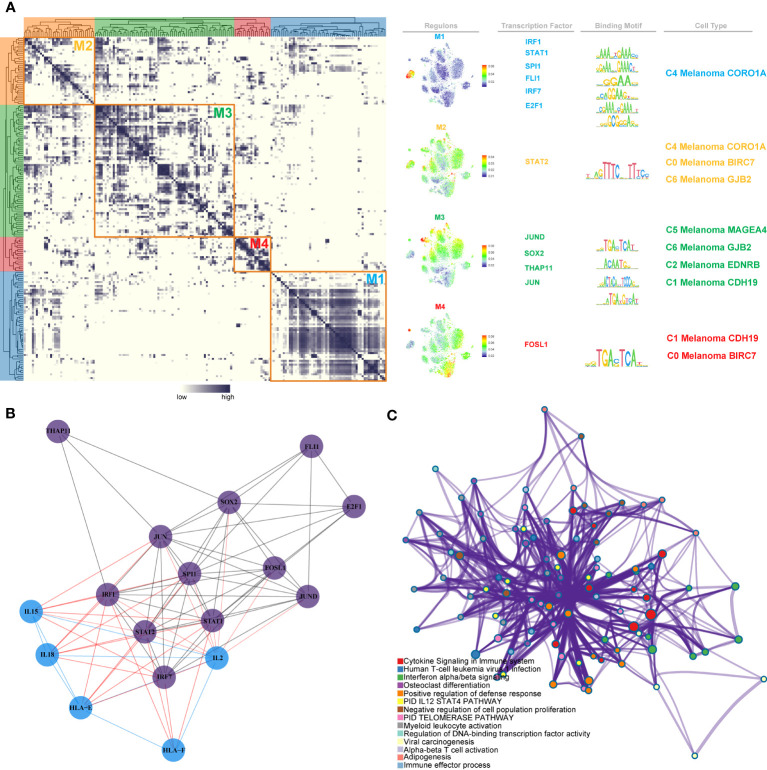
Identification of TF regulon modules in melanoma cell subtypes. **(A)** Based on the connection specificity index (CSI) matrix, identified regulon modules of melanoma cell subtypes, as well as representative transcription factors, related binding motifs, and connected macrophage cell subtypes. **(B)** PPI network of TFs and positive regulation of NK cell activation-related genes. **(C)** GO enrichment analysis of TFs and positive regulation of NK cell activation-related genes.

Furthermore, we discovered regulon modules of melanoma cell subtypes, as well as representative transcription factors, associated binding motifs, and linked macrophage cell subtypes, using the connection specificity index (CSI) matrix. These regulons were grouped into the following 4 major modules (M1, M2, M3, and M4). According to the average activity ratings of each module, the typical TFs and cell types were selected (**Figure 12A**). In addition, the t-SNE plots provided further evidence that the functions of these TFs are highly exclusive to corresponding melanoma cell subtypes. We found that each module occupied a distinct zone and that all highlighted parts exhibited complementary patterns when we mapped the average activity score of each module into t-SNE. The 12 TFs that were inferred to regulate NK and T cells activation in these modules are shown as follows: Module M1 contained the regulators IRF1, STAT1, SPI1, FLI1, IRF7, and E2F1, which are essential regulators for C4 Melanoma CORO1A. Module M2 contained the STAT1 regulator, which has been linked to C4 Melanoma CORO1A, C0 Melanoma BIRC7, and C6 Melanoma GJB2. Module M3 contained JUND, SOX2, THAP11, and JUN, as well as regulators for C5 Melanoma MAGEA4, C6 Melanoma GJB2, C2 melanoma EDNRB, and C1 melanoma CDH19. Module M4 contained regulators such as FOSL1 that are associated with C1 melanoma CDH19 and C0 melanoma BIRC7.

Moreover, a PPI network of these TFs and T and natural killer cell activation-related genes was built. The red lines reflected the interaction between selected genes and TFs associated with NK and T cells activation (**Figure 12B**). Finally, in order to identify the biological significance of these genes and TFs, the GO enrichment analysis was performed (**Figure 12C**).

### Twelve key TFs are involved in the activation of NK and T cells

**Figure 13:**
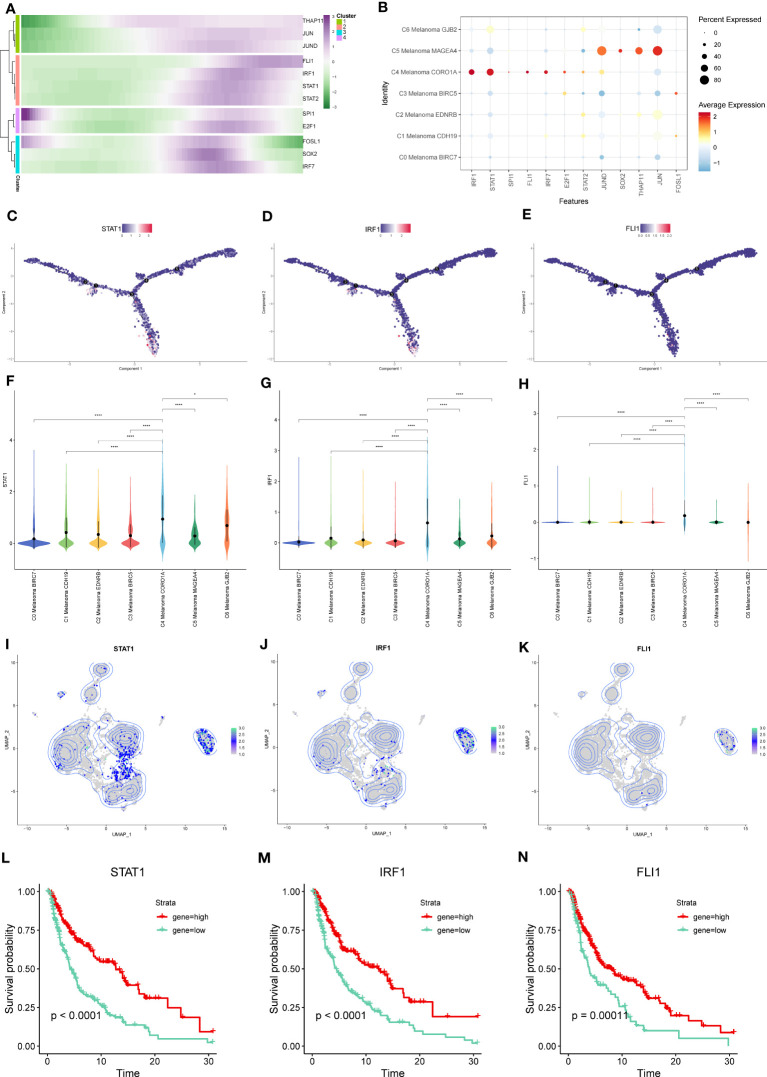
Key TFs of regulon modules in melanoma cell subtypes. **(A)** Heatmap demonstrating the scaled expression of key transcription factors (TFs) in the regulon modules of the melanoma cell subtypes. **(B)** The dot plot shows the expression of these TFs. **(C–E)** The expression of STAT-1, IRF1, and FLI1 in pseudotime trajectories of subtypes of melanoma cells. **(F–K)** The violin plots and UMAP plots show the differential expression of STAT-1, IRF1, and FLI1 among subtypes of melanoma cells. **(L–N)** Kaplan-Meier survival plots of the high and low gene groups for STAT-1, IRF1, and FLI1. *P < 0.05, ****P < 0.0001.

From the heatmap of pseudotime trajectories analysis for these TFs, we found that these TFs were mainly expressed at the ends of pseudotime trajectories, such as THAP11, JUN, JUND, FLI1, IRF1, STAT1, and STAT2 (**Figures 13A, C–E**). In the above TFs, compared to other melanoma subtypes, STAT1, IRF1, and FLI1 were more highly expressed in C4 Melanoma CORO1A (**Figures 13B, F–K**). Interestingly, according to the CSI matrix results, STAT1, IRF1, and FLI1 belonged to Module 1, which primarily regulated gene expression in C4 melanoma CORO1A. In order to further investigate the relationship between gene expression levels of STAT1, IRF1, and FLI1 and survival probability in melanoma patients, Kaplan-Meier survival plots of high and low gene groups for these TFs were utilized (**Figures 13L–N**).

### Kaplan-Meier survival analysis of the core TFs and identification of independent protective or risk factors

**Figure 14:**
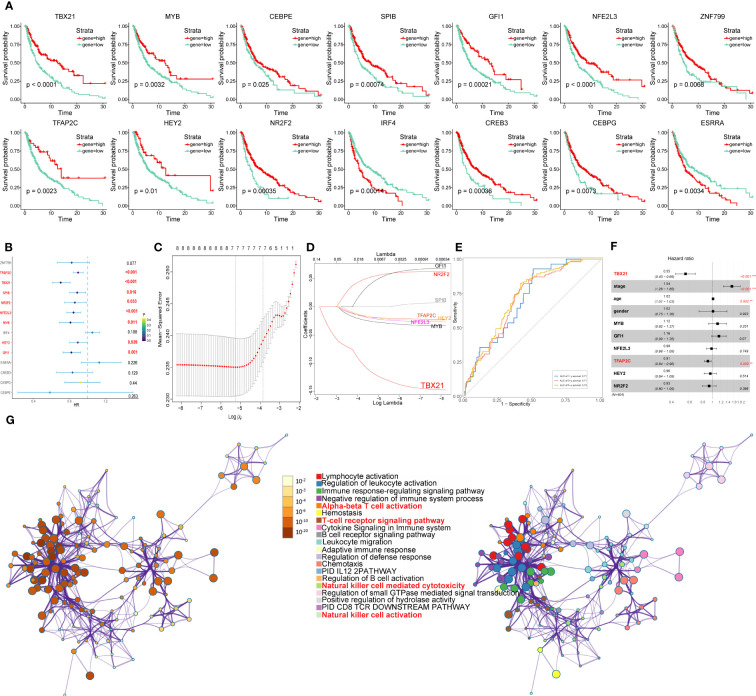
Identification of the overall survival-related TFs. **(A)** Kaplan-Meier survival plots of the high and low gene groups for 14 TFs. **(B)** Univariate Cox analysis of 14 TFs. **(C)** LASSO analysis with 10-fold cross-validation. **(D)** Coefficient profile plots of 8 TFs (TBX21, MYB, GFI1, NFE2L3, TFAP2C, HEY2, NR2F2, SPIB). **(E)** ROC curves of gene signature constructed by 7 TFs (TBX21, MYB, GFI1, NFE2L3, TFAP2C, HEY2, NR2F2) in TCGA- SKCM cohort. **(F)** Multivariate Cox analysis of 7 TFs and clinicopathological characteristics. **(G)** Network of enriched terms colored by cluster identity or p-value, with nodes that share the same cluster identification generally located adjacent to one another.

According to the expression levels of the above core TFs in melanoma cell subtypes, the TCGA-SKCM cohort was divided into two groups (high and low gene expression). Furthermore, in order to investigate the prognostic evaluation value of these core TFs, Kaplan–Meier survival analysis was applied, revealing that 14 core TFs (TBX21, MYB, CEBPE, SPIB, GFI1, NFE2L3, ZNF799, TFAP2C, HEY2, NR2F2, IRF4, CREB3, CEBPG, and ESRRA) were related to the overall survival of melanoma (P<0.05) (**Figure 14A**).

Next, using univariate Cox analysis, 8 TFs (TBX21, MYB, GFI1, NFE2L3, TFAP2C, HEY2, NR2F2, SPIB) associated with OS (P<0.05) were screened and shown as forest maps (**Figure 14B**). LASSO regression analysis was used to filter TFs further in order to identify a biomarker that could predict the prognosis of patients with melanoma (**Figures 14C, D**), based on which seven TFs (TBX21, MYB, GFI1, NFE2L3, TFAP2C, HEY2, NR2F2) were selected. In addition, the ROC curve was used to evaluate the discrimination of gene signature based on 7 TFs, and indicated that the gene signature was highly sensitive and specific depending on the AUC values (**Figure 14E**).

Furthermore, to identify the independent protective or risk TFs of overall survival for patients with melanoma, we applied multivariate COX regression analysis and found that TBX21 is an independent protective factor with HR (0.55, 95%CI: 0.45-0.69) for patients with melanoma (**Figure 14F**).

### Functional enrichment analyses of genes regulated by TBX21

The results of multivariate COX regression analysis revealed TBX21 as an independent protective factor for patients with melanoma. So, in order to further explore TBX21, we applied to GO (performed by Metascape online analyses) of genes regulated by TBX21 and found that the genes were mainly enriched in NK cell-mediated cytotoxicity, NK cell activation, immune response-regulating signaling pathway, and adaptive immune response. Therefore, in order to further investigate TBX21, we used GO (performed by Metascape online analyses) of genes regulated by TBX21 and discovered that the genes were primarily enriched in NK cell-mediated cytotoxicity, NK cell activation, immune response-regulating signaling pathway, adaptive immune response, and so on (**Figure 14G**).

In conclusion, based on the above-reported results, the expression of TBX21 in C4 melanoma CORO1A was significantly different from those of other melanoma subtypes. Furthermore, patients with high expression of TBX21, the most highly regulated transcription factor (TF) of C4 melanoma CORO1A, had higher survival prospects. In order to further investigate the function of TBX21 in melanoma, we performed *in vitro* experiments.

### *In vitro* biological function in melanoma cells

**Figure 15:**
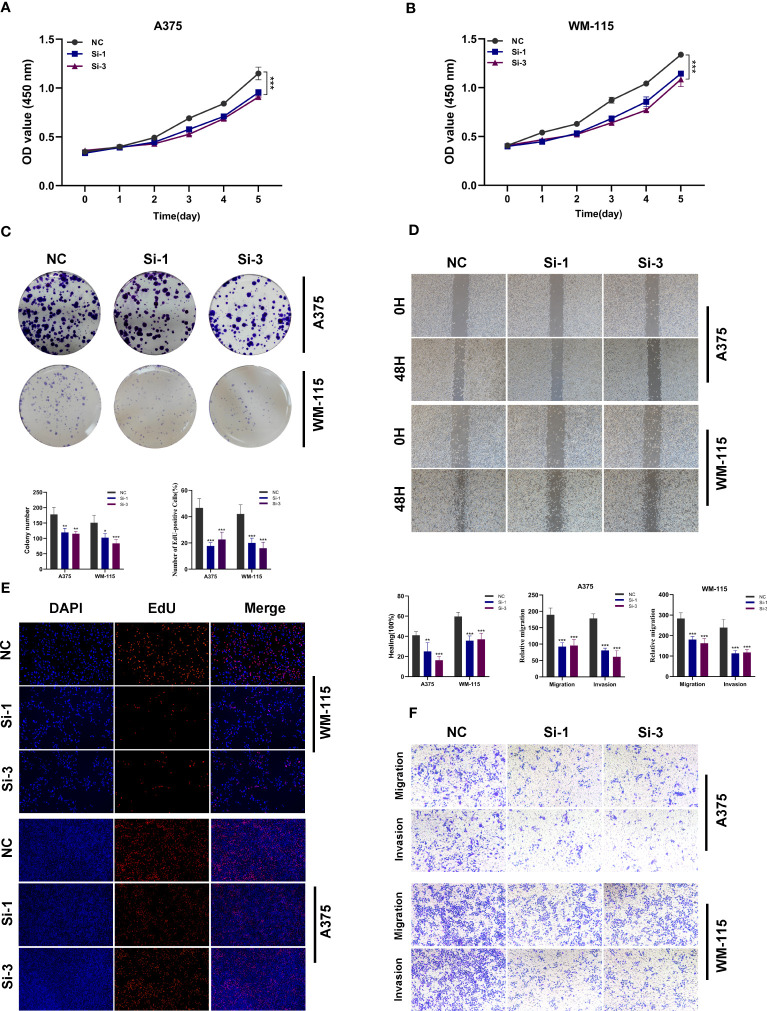
*In vitro* experimental verification of TBX21. **(A, B)** The CCK-8 assay revealed a notable decrease in cell viability following the knockdown of TBX21. **(C)** The colony formation experiment demonstrated that cells with decreased TBX21 expression had a significantly lower number of colonies than the NC group. **(D)** The scratch-wound healing experiment showed that reduced TBX21 expression dramatically decreased the rate of wound healing. **(E)** EdU staining assay demonstrated that the downregulation of TBX21 hindered the proliferation of A375 and WM-115 cells relative to the NC group. **(F)** Transwell experiments showed that the downregulation of TBX21 drastically reduced the migration and invasion of A375 and WM-115 cells. *P < 0.05, **P < 0.01, ***P < 0.001.

In order to further investigate the function of TBX21 in melanoma, we performed *in vitro* tests to further investigate the function of TBX21 in melanoma cells. The level of TBX21 expression 24h after transfection was measured using qRT-PCR to determine the efficacy of siRNA-mediated TBX21 knockdown in A375 and WM-115 cell lines (**Supplemental Figure 2**). The CCK8 test further revealed that after TBX21 knockdown, cell viability decreased significantly in A375 and WM-115 (P < 0.001) (**Figures 15A, B**). The colony formation experiment exhibited that cells exhibiting diminished TBX21 expression displayed a substantially reduced count of colonies in comparison to the NC group (A375 and WM-115 without TBX21 knockdown). Therefore, a slower colony formation rate was seen in TBX21 knockdown cells, suggesting that TBX21 may be essential for the proliferation of the melanoma cell line (**Figure 15C**).

The scratch-wound healing experiment generated equivalent results. Reduced TBX21 expression dramatically decreased the rate of wound healing in cells (**Figure 15D**). Moreover, the findings of the EdU staining assay demonstrated that decreased expression of TBX21 hindered the proliferation of A375 and WM-115 cells relative to the NC group (**Figure 15E**). Transwell experiments demonstrated that TBX21 knockdown drastically reduced the migration and invasion of A375 and WM-115 cells (**Figure 15F**). To illustrate the accuracy and consistency of the results, all tests were performed in two melanoma cell lines (A375 and WM-115), and all data were provided as means standard deviations from three separate experiments. *P < 0.05, **P < 0.01, ***P < 0.001.

## Discussion

Melanoma is usually considered the most lethal kind of skin cancer, mainly due to its high risk of metastasizing and spreading to other organs. In contrast, surgical removal of *in situ* cancers typically has a curative effect. However, managing metastatic disease is more challenging (15, 36, 37). Therefore, the emergence of immunotherapy as a cancer treatment has caused a revolution in oncology and led to adopting new, more effective treatment standards (38–40). Most effective immunotherapy medicines, including monoclonal antibodies, immune checkpoint inhibitors, and chimeric antigen receptors (CARs), target the antigen-dependent adaptive immune system, emphasizing T cells to increase antitumor immunity (41). Since the introduction of ICIs, which reactivate T cell-mediated immune responses against cancer, the survival for metastatic melanoma has improved significantly (42).

Consequently, the vast majority of melanoma immunotherapies have focused on using the adaptive immune system to stimulate the elimination of cancerous cells by B and T lymphocytes (43, 44). Still, as some patients with metastatic melanoma fail to respond to therapy with ICIs, it is urgently needed to explore new treatments to improve the efficiency of therapy and survival of metastatic melanoma. NK cell is a kind of lymphocyte vital to the operation of the innate immune system and is important in the development of the body’s natural defenses against cancer (45, 46).

Additionally, NK cells can be intrinsically activated, meaning they do not require a specific antigen (47). Consequently, in contrast to T lymphocytes, NK cells can eliminate melanoma tumor cells in a special and complementary manner, thus attracting a great deal of interest in the field of immunotherapy in recent years (45, 46, 48). Nevertheless, recent research reported that in metastatic melanoma, NK cells were present at low frequencies, were dysfunctional, and downregulated the expression of TIGIT and CD226 (49). Therefore, exploring the mechanism of NK cell dysfunction in metastatic melanoma is essential.

However, the detailed communication between NK cells and metastatic melanoma cells still remains unclear. Consequently, the activity differences of NK cell-mediated cytotoxicity-related pathways in metastatic melanoma cells are also yet to be elaborated. In order to elucidate the potential communication discrepancies between NK cells and melanoma cells as well as the activity differences of NK cell-mediated cytotoxicity-related pathways in metastatic melanoma tissue, we used the scRNA-seq data of metastasized melanoma. After the QC process, batch effect removal, and initial cell type annotation of 28 clusters, 26,161 cells were grouped into 11 cell types. From the number of cells, the highest proportion of cell types was melanoma cells. Melanoma exhibited the most intratumor heterogeneity (ITH) due to the presence of a large number of clones harboring a range of mutations impacting critical pathways, resulting in abnormally high levels of phenotypic variation and ITH (50), and posing a substantial barrier to customized cancer treatment. However, it remains uncertain to what extent the heterogeneity of melanoma influences the immune microenvironment (51).

To reveal the ITH of melanoma, 10137 melanoma cells were further grouped into seven subtypes (C0 Melanoma BIRC7, C1 Melanoma CDH19, C2 Melanoma EDNRB, C3 Melanoma BIRC5, C4 Melanoma CORO1A, C5 Melanoma MAGEA4, and C6 Melanoma GJB2), each named based on the top marker. Through NK cell-mediated cytotoxicity, NK cells can effectively eliminate multiple types of cancer cells. However, tumor cells can develop resistance mechanisms to circumvent NK cell-mediated elimination (52). The GO enrichment analysis of DEGs in melanoma cell subtypes showed that only the DEGs of C4 Melanoma CORO1A were enriched in NK and T cell-mediated cytotoxicity and related pathways. Thus, we inferred that C4 Melanoma CORO1A might have more sensitivity to NK and T cells compared to other melanoma cell subtypes. In addition, the analysis of AUCell and GSEA was used to further explore the differences in pathway activity among these melanoma cell subtypes. Both indicated the positive regulation of NK and T cell-mediated cytotoxicity and immunity were more activated in C4 Melanoma CORO1A. Moreover, the results of GSEA also revealed that the T cell receptor signaling pathway acquired a higher enrichment score in C4 Melanoma CORO1A than in other subtypes of melanoma.

The aforementioned findings from AUCell, GSEA, and GSVA among melanoma cell subtypes suggested that the C4 Melanoma CORO1A may be more sensitive to NK and T cells by positively regulating NK and T cell-mediated immunity, whereas the other subtypes of melanoma may be more resistant to NK cells. The difference in positive regulation of NK and T cell-mediated cytotoxicity and related pathways between C4 melanoma CORO1A and other melanoma cell subtypes may provide a new perspective that the ITH of melanoma-induced activity and the difference in NK cell-mediated cytotoxicity may have caused NK cell defects. Thus, the potential molecular communication between NK cells and melanoma cell subtypes is vital in uncovering the potential molecular mechanism of NK cell defects in melanoma cells. Interestingly, the overall results of communication networks between NK cells and melanoma cell subtypes suggested that the weights of cell-cell communication between NK cells and C4 Melanoma CORO1A were higher than those of other melanoma cell subtypes, which may lead to the difference in NK cell function between C4 Melanoma CORO1A and other melanoma cell subtypes. Moreover, additional research into outgoing communication revealed that NK cells and C4 Melanoma CORO1A share a similar pattern. NK cells are known to attack MHC-deficient tumor cells because these tumor cells cannot convey inhibitory signals via MHC-specific inhibitory receptors (53). As some of their inhibitory receptors detect MHC-I as ligands, NK cells target tumor cells that have escaped CTL control by downregulating MHC-I expression (54). Interestingly, we discovered that NK cells primarily communicate with C4 melanoma CORO1A via MHC-I. In addition, the Src family kinase LCK can bind to WASH and trigger its phosphorylation during the activation of NK cells, which suggests that Lck may regulate the activity of NK cells (55). These ligand receptors may have a crucial role in NK cell activity in melanoma cells.

In order to explore the key TFs among melanoma cell subtypes, the analysis of gene regulatory networks was used for further research. Four main modules (M1, M2, M3, and M4) were identified among melanoma cell subtypes based on the CSI results. Furthermore, we found that the highest regulon activity scores of melanoma cell subtypes in M1-M4 were C4 Melanoma CORO1A (M1), C4 Melanoma CORO1A (M2), C5 Melanoma MAGEA4 (M3), and C1 Melanoma CDH19 (M4), respectively. Besides, the key TFs of melanoma cell subtypes were identified, including TBX21, the core TF of M1 modules, and C4 Melanoma CORO1A. Through the Kaplan-Meier survival analysis, we found that the high-expression group of TBX21 had a longer survival probability. Interestingly, previous research has found that T-bet (encoded by the TBX21 gene) is essential for NK and T cells’ growth and maturation, and NK and T cells can develop or mature further in the absence of T-bet (56, 57). From another perspective, our study concentrated on the link between melanoma ITH and T/NK cell activity. It is possible that C4 Melanoma CORO1A is more susceptible to NK and T cells than the other melanoma subtypes are. TBX21 is the most important TF in C4 Melanoma CORO1A and is associated with M1 modules. According to the results of SCENIC, the genes predicted to be controlled by TBX21 were utilized for the enrichment of GO. Surprisingly, these genes were mainly enriched for immune response-regulating signaling pathway, T-cell receptor signaling pathway, cytokine signaling in the immune system, adaptive immune response, NK cell-mediated cytotoxicity, and NK cell activation. Thus, we inferred that TBX21 might impact the response of melanoma cells to NK and T cells by regulating the activation of genes associated with NK and T cells.

In our further investigation, we employed the study of gene regulatory networks to examine the important TFs that may regulate the positive regulation of NK and T cells activation-related genes among the melanoma cell subtypes. A total of 12 TFs were identified, among which the expression of STAT1, IRF1, and FLI1 in C4 Melanoma CORO1A was higher than that of other subtypes. In addition, the Kaplan-Meier survival analysis was applied for further investigation, and the results showed that high-expression groups of STAT1, IRF1, and FLI1 had a longer survival probability.

## Conclusion

Based on the single-cell profile of metastatic melanoma, we concluded that C4 Melanoma CORO1A might be more vulnerable to NK and T cells, whereas other subtypes of melanoma may be more resistant to NK and T cells. The difference in NK and T cell-mediated immunity and cytotoxicity between C4 melanoma CORO1A and other melanoma cell subtypes may provide a new perspective that the ITH of melanoma-induced activity and the difference in NK and T cell-mediated immunity and cytotoxicity may have caused NK and T cell defects. Importantly, we discovered that STAT1, IRF1, and FLI1, the protective factors of melanoma, may regulate the response of melanoma cells to NK and T cells by modulating the expression of positive NK and T cell regulation activation-related genes. Although our bioinformatics analysis has yielded valuable insights and we have conducted *in vitro* experimental validation on melanoma cells, the absence of *in vivo* experimental validation limits our ability to confirm the functional implications and mechanisms proposed by our study. Further studies will incorporate *in vivo* experimental validation to further advance our research.

## Data availability statement

The original contributions presented in the study are included in the article/**Supplementary Material**. Further inquiries can be directed to the corresponding authors.

## Ethics statement

Ethical review and approval was not required for the study on human participants in accordance with the local legislation and institutional requirements. Written informed consent for participation was not required for this study in accordance with the national legislation and the institutional requirements.

## Author contributions

ZZ, LL, and GC conceived and designed the research. ZZ, LL, and LT downloaded and collected the data. ZZ and LL analyzed the data and wrote the article. YD performed the experimental part of the paper. LT conducted quality control on the articles. LL and GC guided the submission. ZZ and YD are co-first authors. LL and GC are co-correspondent author. All authors contributed to the article and approved the submitted version.

## Supplementary material

Supplementary Figure 1 AUCell analysis of positive regulation of epithelial-to-mesenchymal transition, oxidative phosphorylation among melanoma cell subtypes. **(A)** Differences in positive regulation of epithelial-to-mesenchymal transition scored per cell by AUCell among melanoma cell subtypes. **(B)** Differences in oxidative phosphorylation scored per cell by AUCell among melanoma cell subtypes. *P < 0.05, **P < 0.01, ***P < 0.001.

Supplementary Figure 2 Cell experiment. **(A)** qRT-PCR to evaluate the level of TBX21 expression in two melanoma (A375 and WM-115) cell lines **(B)** qRT-PCR to evaluate the level of TBX21 expression 5 days after transfection and siRNA sequences could result in significant decrease in TBX21 expression ***P < 0.001.
