# Supplementary figures and images for: Innovative breakthroughs facilitated by single-cell multi-omics: manipulating natural killer cell functionality correlates with a novel subcategory of melanoma cells
Source: Front Immunol. 2023 Jun 26;14:1196892. doi: 10.3389/fimmu.2023.1196892 (PMC10332463; doi:10.3389/fimmu.2023.1196892)

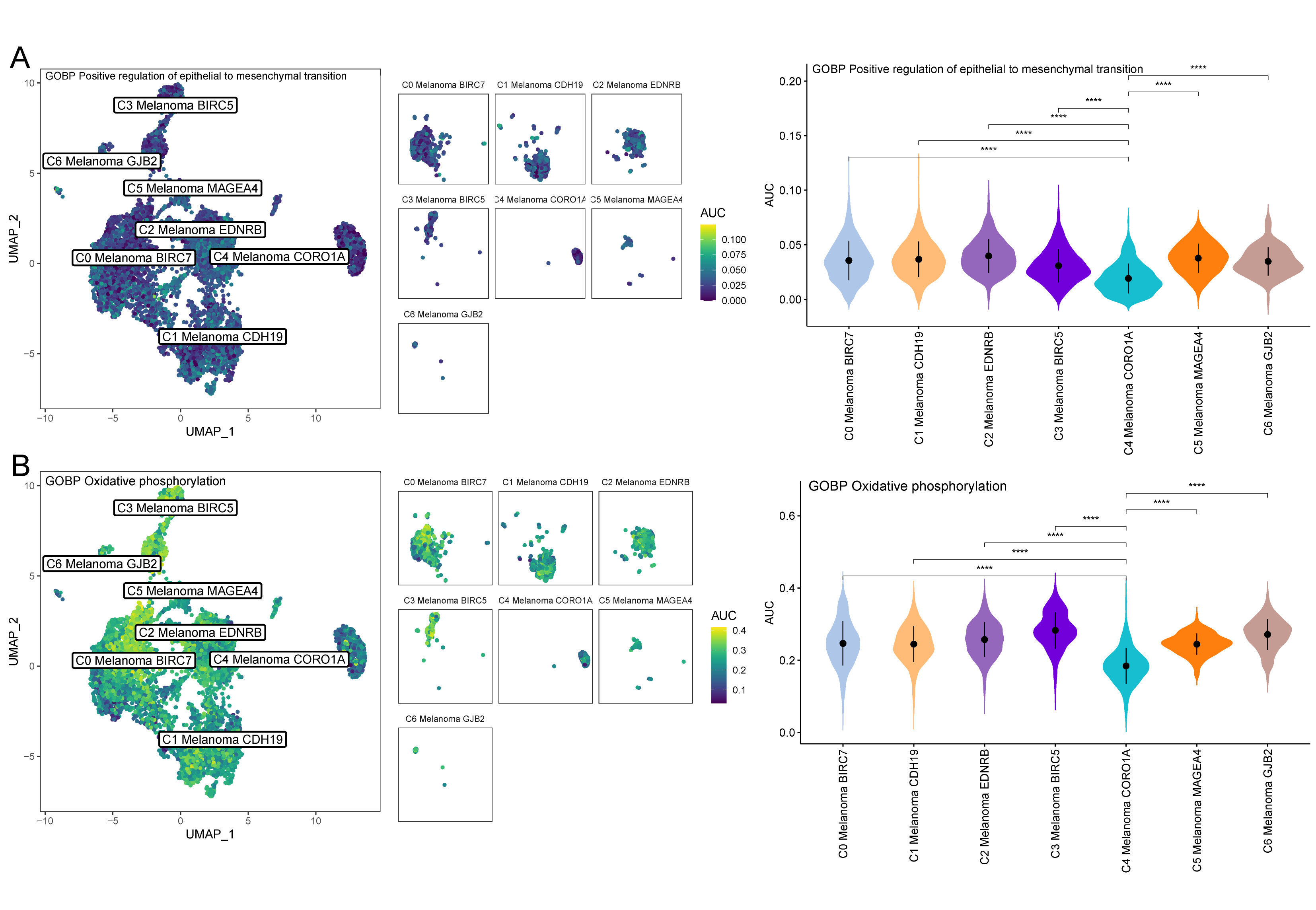

Supplement: Supplementary Figure 1 — AUCell analysis of positive regulation of epithelial-to-mesenchymal transition, oxidative phosphorylation among melanoma cell subtypes. (A) Differences in positive regulation of epithelial-to-mesenchymal transition scored per cell by AUCell among melanoma cell subtypes. (B) Differences in oxidative phosphorylation scored per cell by AUCell among melanoma cell subtypes. *P < 0.05, **P < 0.01, ***P < 0.001. [file Image_1.tif]

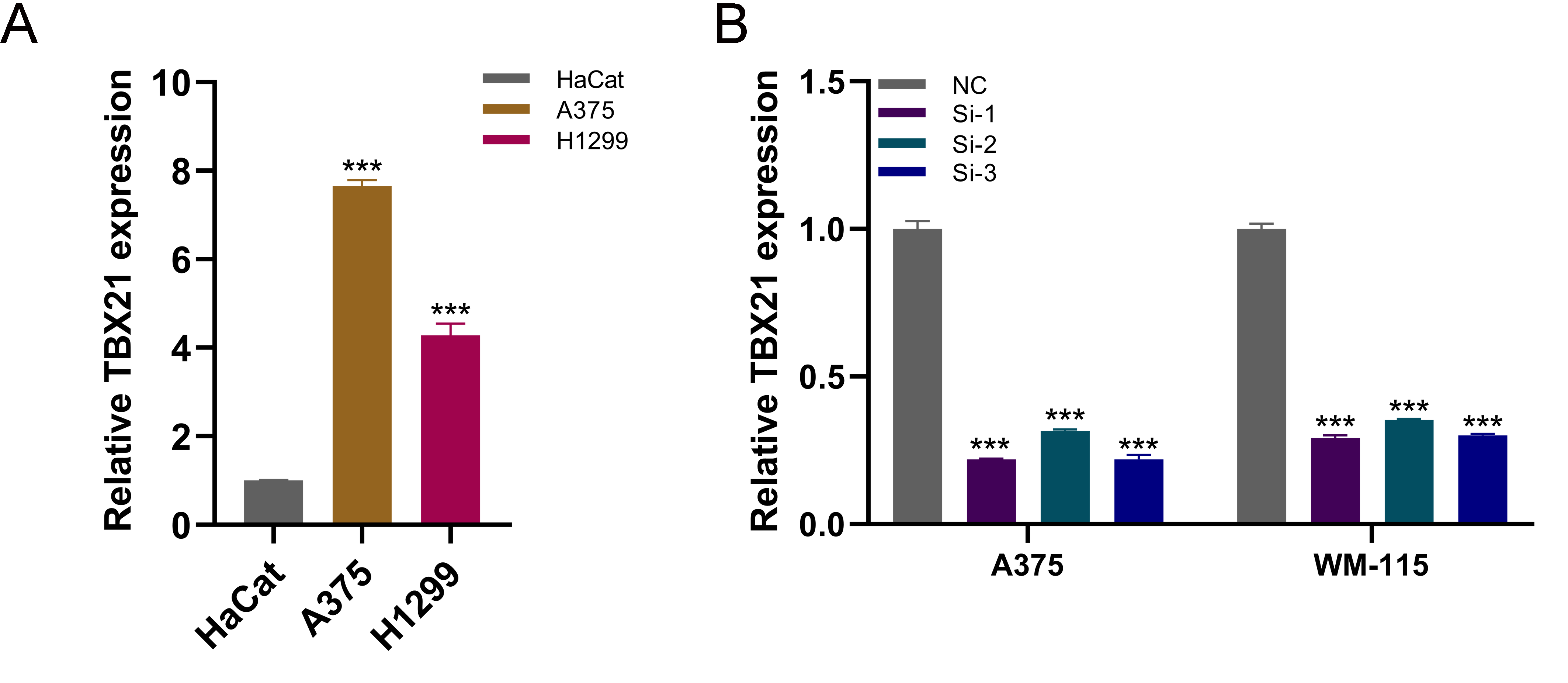

Supplement: Supplementary Figure 2 — Cell experiment. (A) qRT-PCR to evaluate the level of TBX21 expression in two melanoma (A375 and WM-115) cell lines (B) qRT-PCR to evaluate the level of TBX21 expression 5 days after transfection and siRNA sequences could result in significant decrease in TBX21 expression ***P < 0.001. [file Image_2.tif]
