# Supplementary material for: Innovative breakthroughs facilitated by single-cell multi-omics: manipulating natural killer cell functionality correlates with a novel subcategory of melanoma cells
Source: Front Immunol. 2023 Jun 26;14:1196892. doi: 10.3389/fimmu.2023.1196892 (PMC10332463; doi:10.3389/fimmu.2023.1196892)
Supplement: Supplementary file 3 [file Table_1.docx]

| **Oligonucleotides** | **Nucleotide sequence (5'-3')** |
| --- | --- |
| **siRNA** |  |
| Scramble control | GCUUCGCGCCGUAGUCUUA |
| Si-TBX21-1 | CGCTTCCAACACGCATATCTT |
| Si-TBX21-2 | CCAGGAAGTTTCATTTGGGAA |
|  |  |
| **Primer** |  |
| GAPDH | GGCCTCCAAGGAGTAAGACC (forward) |
|  | AGGGGAGATTCAGTGTGGTG (reverse) |
| TBX21 | GCCCACGATGAAACCTGAGA (forward) |
|  | GCTCCTTCATGCCCAAGACT (reverse) |
|  |  |

**Table S1. Oligonucleotides used in research**

**Links to raw data and code**

<https://www.jianguoyun.com/p/DbQcugIQhdXrCBi-y_QEIAA>
